# Assessing the Feasibility and Pre-Post Impact Evaluation of the Beta (Test) Version of the BeUpstanding Champion Toolkit in Reducing Workplace Sitting: Pilot Study

**DOI:** 10.2196/formative.9343

**Published:** 2018-08-28

**Authors:** Genevieve Nissa Healy, Elizabeth G Eakin, Elisabeth AH Winkler, Nyssa Hadgraft, David W Dunstan, Nicholas D Gilson, Ana D Goode

**Affiliations:** ^1^ School of Public Health The University of Queensland Brisbane Australia; ^2^ Baker Heart and Diabetes Institute Melbourne Australia; ^3^ School of Physiotherapy and Exercise Science Curtin University Perth Australia; ^4^ Swinburne University of Technology Melbourne Australia; ^5^ School of Public Health and Preventive Medicine Monash University Melbourne Australia; ^6^ School of Exercise and Nutrition Sciences Deakin University Melbourne Australia; ^7^ School of Sport Science, Exercise and Health The University of Western Australia Perth Australia; ^8^ Mary MacKillop Institute for Health Research Australian Catholic University Melbourne Australia; ^9^ School of Human Movement Studies and Nutrition Sciences The University of Queensland Brisbane Australia

**Keywords:** BeUpstanding Champion Toolkit, implementation, physical activity, sedentary, workplace sitting

## Abstract

**Background:**

The Web-based, evidence-informed *BeUpstanding Champion Toolkit* was developed to provide employers (via a “train-the-champion approach”) with resources and support to help in reducing prolonged sitting in their own desk-based workplace. As part of a five-phase research-to-dissemination process, this study reports on the evaluation of the beta (test) version of this toolkit (Phase 2).

**Objective:**

The objective of our study was to evaluate (1) the implementation of the toolkit by workplace champions and (2) the impact of the toolkit on sitting (primary outcome), standing, and moving; use of activity-promoting strategies; knowledge and attitudes; and indicators of health and work performance.

**Methods:**

An implementation study using a pre-post design was conducted in 7 desk-based workplaces in Australia (September 2015 to May 2016), with work teams (one per workplace) purposively recruited to ensure representation across a range of sectors (white- or blue-collar), organizational sizes (small or medium or large), and locations (metropolitan or regional). All staff within participating teams were invited to participate in the relevant toolkit activities. Implementation outcomes (time commitment required by champions and toolkit activities completed) were collected from each champion via telephone interviews. Changes in impact outcomes, measured via a Web-based questionnaire completed by employees at baseline and 3 months postimplementation, were assessed using mixed models, correcting for clustering.

**Results:**

Champions reported a 30-60 minutes per week time commitment to the toolkit activities. All teams formed a wellbeing committee and sent the staff surveys at both time points; most champions held a staff consultation workshop (6/7), identified team-level strategies within that workshop (5/7), used the communication resources provided within the toolkit (emails, posters; 6/7), and completed the action plan (5/7). In total, 52% (315 of ≈600) employees participated in at least one survey and 97 (16%) participated in both. At follow-up, there was a significant (*P*<.05) reduction in self-reported workplace sitting time compared to baseline (−6.3%, 95% CI −10.1 to −2.5; n=85) equating to ≈30 minutes per workday. Significant benefits were also observed for the use of activity-promoting strategies, with small, nonsignificant changes observed for knowledge and attitudes and indicators of health and work performance.

**Conclusions:**

The beta version of the *BeUpstanding Champion Toolkit* was feasible to implement and effective in reducing self-reported workplace sitting across a broad range of desk-based workplaces. The next phase (Phase 3) will build on these findings to optimize the toolkit for wider-scale implementation and longer term evaluation.

## Introduction

Regular participation in moderate-to-vigorous physical activity (MVPA) has long been the cornerstone of chronic disease prevention efforts. However, in Australia, population participation levels of leisure time MVPA are low and have been relatively unchanged for 22 years, despite associated public health efforts [1]. More recently, the relevance of time spent in nonexercise activities, such as sitting and light intensity activities, for indicators of health and wellbeing has been increasingly recognized. Excessive sitting is now acknowledged as a probable contributor to the risk of major chronic diseases (type 2 diabetes and cardiovascular disease in particular [2]). Notably, a meta-analysis using data from over one million adults reported that only very high volumes of MVPA (≥60 minutes per day) seemed to eliminate the risk of death associated with high sitting time [3]. As such, public health guidelines now recommend a dual message of moving more and sitting less [4].

One of the key settings to address excessive sitting time is the desk-based workplace [5]. On average, desk-based workers spend 75% of their work day sitting, with much of this time accrued in prolonged, unbroken bouts of ≥30 minutes [6,7]—a pattern that may be particularly harmful to indicators of cardiometabolic health [8]. As such, desk-based workers have been identified as a large and growing at-risk population subgroup [9,10]. The relevance of addressing workplace sitting time for workplace health and safety [10,11] and for public health [12] has been acknowledged, and there have been several recent interventions, incorporating a range of activity-promoting strategies, which have demonstrated that reducing prolonged sitting is feasible and acceptable to both employers and employees within the desk-based workplace [6,7,13-15]. Many, but not all, interventions are also effective [16,17].

To provide employers with the resources and support to translate this research evidence into practice, we developed the *BeUpstanding Champion Toolkit.* This free, Web-based toolkit provides an evidence-informed, step-by-step guide with accompanying resources to help work teams create a dynamic work environment where standing up, sitting less, and moving more is the norm. The *BeUpstanding* program delivered through the toolkit is primarily based on the interventions developed, and evidence generated, from the *Stand Up Australia* program of research [6,7,13-15,18-22]—a program that targeted multiple levels of influence (organizational, environmental, individual, or combinations of these) to address reductions in prolonged workplace sitting time [7]. The flagship of this program was the *Stand Up Australia* intervention [18], which was shown to be strongly efficacious for reducing workplace sitting time within the context of a cluster randomized controlled trial [7]. The key adaptation from *Stand Up Australia* for *BeUpstanding* was the transfer of intervention implementation and evaluation from the research team to a workplace champion. This “train-the-champion” approach recognizes that workplace champions, as the role models and drivers, are critical for successful and sustainable workplace change [23-25].

The translation of the *Stand Up Australia* intervention program into a scalable and sustainable workplace health and safety program involves multiple phases [26]. Phase 1, which has been completed, involved the initial development of the toolkit and formation of research-government partnerships. A detailed description of this development has been described elsewhere [26]. The current paper concerns Phase 2, in which the beta (ie, test) version of the toolkit was piloted among a small, diverse set of workplaces. This pilot enabled us to evaluate whether the Web-delivered “train-the-champion” approach, as a potentially scalable way to deliver *Stand Up Australia*, was feasible to implement by workplace champions. As noted above, the interventions that informed *BeUpstanding* have undergone rigorous evaluation and have demonstrated strong efficacy for workplace sitting reduction [6,7,13-15]. The impact evaluation within this pilot study was intended to provide some corroborative evidence about the likely effectiveness of the intervention following the adaptations made for scalable delivery, as well as explore relevant measurement issues (responsiveness to change and likely effect sizes). The specific aims were to evaluate (1) the implementation of the toolkit by the workplace champions and (2) the impact of the toolkit on sitting (primary outcome), standing, and moving; use of activity-promoting strategies; knowledge and attitudes; and indicators of health and work performance. The findings from this phase of research will inform the optimization (Phase 3) of the *BeUpstanding Champion Toolkit* prior to any wide-scale implementation and evaluation.

## Methods

### Study Design and Recruitment

The evaluation used a pre-post design. A key limitation of the previous trials evaluating the *Stand Up Australia* intervention is the limited diversity of workplaces (typically, white-collar employees of reasonably large metropolitan organizations have been represented) [7,14,15]. Accordingly, the evaluation used purposive sampling to ensure coverage of desk-based workers from a range of industries (including white- or blue-collar), organizational sizes (small or medium or large, ie, <20/20-500/>500), and locations (metropolitan or regional). The sample size of 7 work teams was selected to cover the range of desired workplace attributes; it was not selected *a priori* based on the requirements of the impact evaluation. Over a two-month period, workplace representatives were made aware of the study by project staff (who extended personal invitations to existing contacts and delivered seminars to workplace wellness networks) and by the project’s government partners (who used a variety of promotional endeavors), with workplaces selected from those who expressed interest. While the workplaces were selected purposively, the project staff had no input concerning the selection of teams within workplaces (one per workplace), nor champions for teams. A senior project manager guided workplace champions through the consent stage and provided access to the beta version of the toolkit via a password-restricted login page. This manager was also available throughout the study to answer questions as required. All employees within the participating work team were provided information on the program, were exposed to program messaging (via the champion), and were invited to take part in the staff consultation workshop and Web-based surveys (see below). The study was approved by the Behavioral and Social Sciences Ethical Review Committee of the University of Queensland, with champions providing written informed consent and staff providing informed consent prior to participating in any data collection.

### The *BeUpstanding Champion Toolkit*: Beta (Test) Version

**Figure 1:**
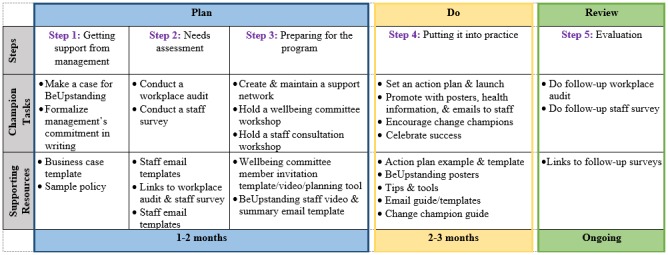
BeUpstanding Champion Toolkit steps, tasks, supporting resources, and timeline.

The Web-based *BeUpstanding Champion Toolkit* provides a step-by-step guide to support workplace champions to adopt, deliver, and evaluate the program within their own work teams. The structure of the toolkit and the associated resources are detailed in Figure 1, with the 5 steps following the “Plan, Do, Review” phases of the Work Health and Wellbeing Framework [27]. The Plan phase involves obtaining support from management (step 1), conducting a needs assessment (step 2), and preparing for the program (step 3). The Do phase involves putting the program into practice (step 4), while the Review phase involves evaluation of the impact of the program on both policy and practice (step 5). Each step includes an instructional component explaining the purpose of the step (ie, to “train” the champion). The activities within the toolkit are intended to be implemented over a three-month period at the level of the work team (broadly defined as a colocated group, employed by the same organization, and having the same workplace policies). Resources within the toolkit are mixed media, including editable word and email templates, PDF posters and tips sheets, and videos. These supporting materials were designed to help initiate change through increasing awareness (eg, email templates, posters), as well as create longer term organizational level change through building a supportive work culture and environment (eg, sample policy statements, workplace audits).

Core tasks supported by the toolkit include forming a wellbeing committee and holding a wellbeing committee workshop to plan the initiative; holding a staff consultation session to educate staff and collectively, through a participative approach, identify 3 top strategies to stand up, sit less, and move more; and implementing and promoting these strategies across the work team (Figure 1, Steps 3 and 4). The accompanying resources for the workshops included in the toolkit emphasize selecting strategies to increase standing or moving (predominantly light intensity activities) that target work practices within a team (eg, standing meetings) or the work environment (eg, centralizing printers) to encourage sustainable organizational level change. Suggestions were provided within the toolkit for the wellbeing committee concerning strategies that may be able to be implemented immediately (including low-cost or no-cost strategies) as well as those that may require longer term planning or resourcing, with teams also encouraged to brainstorm their own strategies that best suited their team.

### Data Collection and Measures

#### Characteristics of Workplaces, Champions, and Staff

Data on the workplaces (size, location, sector), as well as reasons for taking part in the program, were collected via the initial expression of interest and confirmed with champions. Information on the job role of the champion was collected as part of the initial contact by the senior project manager.

Anonymous Web-based surveys were used at baseline and 3 months postimplementation to collect self-reported data regarding the primary and secondary impact outcomes as well as age, gender, work hours, job role (management, general staff, other), and education (some high school, completed high school, post high school). Participants were also asked the number of days in the last week where they had done a total of ≥30 minutes of physical activity that was enough to raise their breathing rate [28]. The responses were used to identify the participants who met minimal physical activity guidelines (ie, ≥30 minutes moderate activity for ≥5 days per week) [4]. Satisfaction with the program was assessed in the postimplementation survey only (single-item, 10-point scale; 10 = “very satisfied”). The participant responses to 3 questions generated an identity code that was used to match pre- and postintervention survey data to the same (anonymous) participant. A cross-check of the gender and date of birth of “new” respondents at the postintervention survey against possible partial matches from the baseline survey was used to identify failures in the matching process (n=3 participants) and rectify the data accordingly.

#### Implementation Measures

Implementation data were collected via telephone interviews with the champions following the completion of Step 5 of the program described in Figure 1 (after approximately 3 months of implementation). A checklist was used to collect data on the completion of the steps in the program and the use of the accompanying resources, as well as the estimated time commitment by the champion for program implementation. This information was used to inform the feasibility of implementation.

#### Impact Outcomes

##### Sitting, Standing, and Moving

The primary impact outcome was self-reported workplace sitting time. The secondary activity-related outcomes were as follows: workplace time spent standing, walking, in heavy labor, and moving (walking + heavy labor); workplace sitting accumulation; and time before and after work and on nonwork days spent sitting, standing, and moving.

Behaviors at work were measured by the Occupational Sitting and Physical Activity Questionnaire [29], which asks about the percentage of time on a typical day in the last 7 days spent sitting, standing, walking, or in heavy labor or physically demanding tasks. This questionnaire has acceptable validity and reliability compared with posture-based activity monitors [30] and is responsive to change [30]. In addition to asking about work hours, the questionnaire was further adapted to measure these activities outside of worktime on work days (ie, *“How would you describe the time you were awake before and after work on a typical work day in the last 7* *days* *?”)* and nonwork days. All questions referred only to times the participants were awake.

Participants were also asked how many breaks from sitting (0-5 or more) they typically took in an hour while at work [31]; the longest period they spent without getting up at work on a typical work day in the last 7 days; and the percentage of their workplace sitting time that involved sitting for long periods at a time (≥30 minutes continuously) and conversely sitting in an “interrupted” manner (<30 minutes), with the sum total of their 2 responses being 100% of workplace sitting time. The last 2 questions were new questions developed for this study.

##### Knowledge and Attitudes Toward Sitting, Standing, and Moving

Baseline awareness of the health impacts of “too much sitting” was assessed by an open-ended question in the staff survey. Participants were asked to list all the health impacts of sitting of which they were aware. These response options were then coded into mutually exclusive categories concerning plausible benefits (5 categories: musculoskeletal; weight gain or obesity; cardiovascular health or diabetes or metabolism; other vascular (eg, circulation); and fatigue or concentration), “other”, “not sure”, and “none”. Change in knowledge was assessed via a knowledge score. Participants were asked “*How often do you think you should get up from sitting?* ” with response options of *“Every? hours”* or *“Every? minutes* *”.* One point was assigned for every 5 minutes that their response deviated from the message provided in the *BeUpstanding* program (at least every 30 minutes). Responses of 25-35 minutes were treated as correct (0 points), with the lowest scores indicating the best knowledge.

The impact on attitudes was assessed in terms of the following: desired activity at work; the difference between desired and performed behavior; control over sitting and standing at work; and workplace support for sitting and standing. Through the question “*If you were given a choice at work, what percentage of the time would you spend…,”* participants were asked about their desired sitting, standing, and moving at work (scores were required to add up to 100% of the time). Gap scores were then calculated as the absolute value of the difference between self-reported desired and performed activity at work. In theory, gap scores range from 0 (desired and performed are exactly the same) to 100 (desiring 100% and doing 0%, or vice versa). Control over sitting and standing at work, and workplace support for sitting and standing, were assessed by participant responses concerning the extent to which the participants agreed with the two statements: *“I have control over whether I sit or stand at work”* and *“My workplace is committed to supporting staff choices to sit or stand at work”* [32]. Both questions used a five-point Likert scale (strongly disagree, disagree, not sure, agree, or strongly agree).

##### Activity-Promoting Strategies

Participants were provided with a menu of 13 common strategies that have been used to promote standing up, sitting less, and moving more in the desk-based environment [6,33,34] and were then asked to indicate the extent to which they used these strategies (never or rarely, sometimes, often or very often, not applicable). Strategy use was measured as the percentage of the 13 strategies participants reported using at least “sometimes”.

##### Work Performance Indicators and Perceived Health Status

Work performance was measured by a 9-item, 10-point scale [35] with findings reported as the mean of the 9 items. Job satisfaction in the past week was measured by a single-item, 10-point scale (1 = very dissatisfied; 10 = very satisfied) [36]. Current energy and stress levels were measured on a single four-point scale (1 = “I always have lots of energy”; 4 = “I feel exhausted most of the time” and 1 = “I don’t feel unduly stressed most of the time”; 4 = “I feel under incredible stress most of the time”), with energy and stress scores reversed such that a higher score indicated more energy or less stress. Self-rated health in the past week was measured on a single, five-point scale (1 = poor; 5 = excellent) that has shown strong correlation with the World Health Organization self-rated health measure [37].

### Analyses

Data were analyzed in SPSS (version 24, IBM Corp., Armonk, NY, USA) and STATA version 14 (StataCorp, College Station Texas, TX, USA). Descriptive data are reported as mean (SD), median (25th, 75th percentile), or n (%). Findings are reported in terms of the effect size and statistical significance, with significance set at two-tailed *P*<.05. Minimum differences of interest (MDI) for the activity measures were set at 30 minutes of sitting and standing or 10 minutes of walking, heavy labor, or moving per workday or per day (ie, 6.25% of work hours, 3.125% of waking hours based on a theoretical 8-hour workday and 16 hours awake). The 30-minute MDI for sitting and standing is consistent with a single bout of prolonged sitting time (30 minutes) and is in between the MDIs used in Stand Up Victoria of 45 minutes [32] and average sedentary reductions in intervention studies (approximately 20 minutes) [16]. Ten minutes is the smallest period of activity that self-report questionnaires, such as the Active Australia, typically require participants to recall [38]. For other outcomes, MDI was set at changes consistent with a “small” effect (0.2 SDs), with changes less than the MDI considered very small.

Changes over time in the primary and secondary impact outcomes (all continuous) were tested using STATA’s mixed procedure, using random terms for the repeated measures and site-level clustering. To yield insight concerning which workplaces may benefit most or least (in terms of workplace sitting time) from the intervention when upscaling the intervention, mixed models were used to explore workplace variation in change. The main analyses for the primary and secondary outcomes were evaluable case analyses. To assess the sensitivity of conclusions to missing data, the primary outcome (workplace sitting) was re-evaluated using multiple imputations by chained equations, with a mean of 80 imputations used in view of the fraction of missing information. Other analyses corrected for clustering via linearized variance estimation (survey commands). Not all questions were compulsory; hence, the number of respondents varies depending on the question. Sociodemographic predictors of survey completion or noncompletion were tested using linear or logistic regression. To inform future evaluations of *BeUpstanding* and similar interventions, we report on the extent of clustering encountered and the responsiveness to change of each of the outcome measurements, that is, standardized effect size (SES) and standardized response mean (SRM). SES was calculated as mean change divided by SD at baseline, while SRM was calculated as change divided by SD of change with mean and SD of change scores calculated using linearized variance estimation. Larger absolute values of SES or SRM indicate better responsiveness to change.

## Results

### Characteristics of Participating Workplaces, Champions, and Staff

#### Workplace Characteristics

The 7 teams of desk-based workers were recruited across organizations that were small (Team A), medium (Teams B, D, F, and G), or large (Teams C and E; Table 1). Industries with primarily blue-collar work were represented by Teams B and G. In total, 4 of the work teams were from governmental organizations (Teams C, D, E, and F) and the remaining were from the private sector. Of the workplaces, 2 (Workplaces C and F) already had sit-stand workstations installed. The reasons champions provided for taking part in the intervention were consistent across work teams, with all indicating that they anticipated the program would help create a healthier workplace. Furthermore, 6 workplaces mentioned that they expected the program would change awareness, culture, or practices around excessive sitting.

#### Champion Characteristics

A total of 7 workplace champions (4 women, 3 men) delivered the *BeUpstanding* program, with 6 of the champions employed in a managerial or senior leadership role (Table 1).

#### Staff Characteristics

In total, approximately 600 staff were exposed to the program with 315 workers participating in at least 1 survey, 237 workers responding to the baseline survey, 170 responding to the follow-up survey, and 97 workers responding (at least partially) to both surveys. Participation rates, that is, the total number of respondents to either survey divided by the work team size as reported by the champion, ranged from 37% to 100% across the work teams, with approximately 52% participation overall (Table 1). Table 2 shows the participants’ sociodemographic and work-related characteristics. They had an average age of approximately 40 years and work hours consistent with full-time work. Most of the sample were women (65%), had a university education (72%), and described their job role as management (78%).

Approximately a third of participants reported meeting the physical activity guidelines. Although nonparticipants were not assessed, a comparison of those who completed only one survey, as opposed to both, suggested that participation biases were minimal. Compared with their counterparts, those who completed 1 survey were significantly younger (*P*=.04; mean difference=−3.7 years; 95% CI: −7.1 to −0.4) but were otherwise similar in terms of gender, education, job role, work hours, and meeting minimal physical activity guidelines (all differences were nonsignificant and <10%).

**Table 1:** Baseline characteristics of work teams and champions participating in the pilot study.

Work place	Industry	Location	Reasons for taking part	Approximate work team size, n	Survey participation rate^a^, n (%)	Champion job role	Wellbeing committee, n
A	Engineering	Regional	Hoping to get everyone in the office moving a little more and being mindful about their health and how much time they’re spending in a chair.	12	9 (75.0)	Receptionist	4
B	Plumbing	Outer region of CBD^b^	Explore options for us to lead the way in creating a healthier workplace.	16	16 (100.0)	Corporate Services Manager	3
C	Public administration	CBD	Improved employee health, work culture, and practices.	300	117 (39.0)	Rehabilitation and Employee Relations Case Manager	8
D	Public administration	Outer region of CBD	Assistance to make my workplace healthier in order to help staff and the organization.	40	27 (67.5)	Assistant Principal Officer	6
E	Public administration	CBD	We hope to improve the way we promote and sustain better health for our staff making use of the research outcomes (learning and aids) of the program developers and the research outcomes of the pilot to provide a healthy workplace that staff are interested in being part of. This should then lead to people wanting to work here (our division), and for those already here, to their achievement of improved outcomes.	100	74 (74.0)	Principal Governance and Improvement Officer	7
F	Workplace policy regulation	CBD	To raise awareness, educate staff, change current sedentary work practices, and contribute positively to the long-term health of our staff.	100	54 (54.0)	Acting Government Solicitor and Senior Procurement and Contracting Officer	5
G	Transportation and logistics	Outer region of CBD	A change in the consistent sitting in the office. A change in thinking around being tied to the desk other than break times. Regular standing breaks and use of the stairs. An overall improvement in the sense of wellbeing of all desk-based staff.	35	18 (51.4)	Customer Support Manager	4

^a^Number of respondents to one or both surveys and approximate workplace size; an indicator of the “reach” of the intervention.

^b^CBD: central business district. Outer region of CBD: >15 km from CBD.

**Table 2:** Characteristics of participants who completed the Web-based surveys.

Characteristics	Either survey	Both surveys^a^	Baseline survey only	Follow-up survey only
Number of particiants, n	315	97	237	172
Age in years, mean (SD)	39.7 (11.5)	42.3 (11.1)	40.0 (11.3)	42.3 (11.1)
Work (h/wk), mean (SD)	38.2 (6.3)	38.1 (5.3)	38.2 (6.5)	38.5 (6.1)
Women, n (%)	202/311^b^ (65.0)	64/96 (66.7)	154/236 (65.3)	112/171 (65.5)
University education, n (%)	223/311 (71.7)	66/96 (68.8)	173/236 (73.3)	120/170 (70.6)
Management job role, n (%)	241/311 (77.5)	73/96 (76.0)	179/236 (75.9)	135/170 (79.4)
Met minimum physical activity guidelines of ≥30 min moderate activity for ≥5 d/wk), n (%)	83/294 (32.3)	31/96 (32.3)	64/215 (29.8)	46/161 (28.6)

^a^As reported in the baseline survey.

^b^Number of participants vary as not all particpants provided information pertaining to the characteristics listed.

### Aim 1: Evaluation of the Implementation of the *BeUpstanding* Program

Table 3 provides details on the implementation checklist according to work team. All champions made the program available to all staff within their work team, all teams formed a wellbeing committee to identify potential strategies, and all champions sent the surveys (baseline and follow-up) to all staff within their team. Most champions held a staff consultation workshop (6/7), identified strategies within that workshop (5/7), used the communication resources provided within the toolkit (emails, posters; 6/7), and completed the action plan (5/7). When asked how much time on average they spent working on the program, the majority of champions indicated between 30 minutes and 1 hour per week, with more time spent initially (ie, the first month). This initial commitment varied from 1 to 2 hours for most work teams to 1 day per week for the first month for the largest workplace (Team C), where multiple staff consultation sessions were conducted by the champion.

### Work Team Strategies to Stand Up, Sit Less, and Move More

A broad range of standing and movement strategies were chosen by the work teams as part of the staff consultation workshop (Table 4). Notably, 2 of the work teams did not choose any strategies (A and E); 2 (C and D) made further divisions within their team, with strategies identified for each of these “subteams” (Teams C and D had 6 and 3 subteams, respectively); and 1 (G) chose approximately 30 strategies, with a different staff member responsible for leading one of the strategies each day. Common strategies chosen by work teams included standing phone calls, taking the longer route to a destination, walking meetings, centralizing printers or bins, and the use of prompts and reminders. Unique strategies included dance-offs and stepathon competitions (ie, the accumulation of steps over a time period).

### Aim 2: Evaluation of the Impact of the *BeUpstanding* Program

#### Prior to *BeUpstanding*

At baseline, participants showed some awareness of the potential health effects of excessive sitting, with 170 (n=216) listing at least one impact, while 43 indicated that they were “not sure” of any health impacts and 3 stated there were no health impacts. Of the 170 participants who listed at least one impact (376 impacts in total), musculoskeletal effects were the most commonly identified (128/376, 34.0% of responses), followed by cardiometabolic (which included cardiovascular health, diabetes, and metabolism; 70/376, 18.6%), weight gain or obesity (60/376, 16.0%), other vascular issues (50/376, 13.3%), and fatigue or concentration (24/376, 6.4%). The diverse range of responses grouped as “other” (44/376, 11.7%) included impacts relating to cancer, stress, fatty liver, and digestion. All responses given were broadly plausible given the evidence, though some were nonspecific (eg, negative impact on health and wellbeing) or indirect (eg, loss of fitness).

Table 5 shows the average levels of each primary and secondary outcome among those participating in the baseline survey (n=212-218; baseline data from evaluable participants who participated in both surveys is presented in Multimedia Appendix 1). Prior to the intervention, on average, most workplace time was spent sitting (mean 78.6% [SD 15.7%]), while less time was spent standing (mean 11.6% [SD 12.5%]) or moving (ie, walking or engaging in heavy labor; mean 9.8% [SD 7.6%]). Furthermore, the reported accumulation of workplace sitting was consistent with sitting for long periods at a time, with an average of 71.1% (SD 20.2%) of sitting time reportedly accrued in a prolonged unbroken manner and with the longest period of continuous sitting over the last week averaging 138.1 (SD 62.2) minutes.

**Table 3:** How many and which teams implemented each implementation checklist item.

Implementation checklist item	Work team							Frequency (N=7), n (%)
	A	B	C	D	E	F	G	
Was the program made available to all staff?	✓	✓	✓	✓	✓	✓	✓	7 (100)
Did you form a wellbeing committee?	✓	✓	✓	✓	✓	✓	✓	7 (100)
Did the wellbeing committee attend the committee information workshop?	✓	✓	✓	✓	✓	✓	✓	7 (100)
Did the wellbeing committee watch the committee information video?		✓	✓	✓		✓		4 (57)
Was the wellbeing committee involved in the identification of strategies?	✓	✓	✓	✓	✓	✓	✓	7 (100)
Was a staff consultation workshop held?	✓	✓	✓	✓		✓	✓	6 (86)
Was the staff information video played?			✓	✓		✓		3 (43)
Did staff identify top strategies at the workshop to do as a team?		✓	✓	✓		✓	✓	5 (71)
Was an action plan template completed?	✓	✓	✓			✓	✓	5 (71)
Were posters placed around the office?	✓		✓	✓	✓	✓	✓	6 (86)
Were program information emails/newsletters sent to staff?	✓	✓	✓		✓	✓	✓	6 (86)
Were all staff sent the link to the online staff survey at baseline?	✓	✓	✓	✓	✓	✓	✓	7 (100)
Were all staff sent the link to the online staff survey at follow-up?	✓	✓	✓	✓	✓	✓	✓	7 (100)

**Table 4:** Strategies chosen at the staff consultation workshops to Stand Up, Sit Less, and Move More.

Work team^a^ and subteam		Strategies
Work team B		Standing meetings Walking teams Removed extra chairs from champion’s office Stretching sessions Encourage face-to-face meetings internally rather than just picking up the phone Group activities for staff outside of work hours that encourage movement like bowling and mini golf
**Work team C**		
	Subteam 1	Centralized recycling bins Outlook calendar reminder to stand every 30 min Take the longer route
	Subteam 2	Outlook calendar reminder to stand every 30 min Walk and talk Use alternative printer that is further away
	Subteam 3	Standing phone calls and greetings Drink more water Active breaks away from desk
	Subteam 4	Outlook calendar reminder to stand every 30 min Walk and talk Drink more water
	Subteam 5	Standing phone calls Centralized printer Deliver and collect mail
	Subteam 6	Fill your own water bottle – take the long route Walk and talk Centralized printer
**Work team D**		
	Subteam 1	11 am and 2:30 pm team stretch Stand up when you hang up Drink more water
	Subteam 2	Stand and stretch to welcome the last person in Walk to talk to colleagues Take breaks outside
	Subteam 3	Collect and deliver mail Remove the mail trays Rhythm and Blues Friday dance-offs
Work team F		10 at 10 – email every day to stand up and stretch at 10 am Standing meetings Stepathon
Work team G^b^		Break up sitting every hour by doing squats, funny dances Having a “wear your sneakers to work” day At lunch, doing a 20-minute walk and 10 minutes of eating

^a^There were no specific staff-level strategies chosen by Workplace A or Workplace E.

^b^30 strategies put forward: participants took part in the “strategy of the day” (listed on calendar). Strategies listed for this workteam showcases examples of "strategy of the day."

**Table 5:** Baseline levels and changes in primary and secondary impact outcomes following *BeUpstanding*. Mean and SD corrected for clustering (linearized variance estimation) at baseline in baseline survey respondents and mean change (95% CI) and *P* values reported for difference from mixed models in, within those reporting outcome data and follow-up survey respondents (n=80-85 depending on the outcome).

Impact outcomes		Baseline (n=218)		Evaluable cases (n=85)		
		n^a^	Mean (SD)	n^b^	Change (95% CI)	*P* value
**Work activity, % of work time**						
	Sitting	218	78.6 (15.7)	85	−6.27 (−10.08 to −2.46)^c^	.001^d^
	Standing	218	11.6 (12.5)	85	2.33 (−0.55 to 5.21)	.11
	Moving^e^	218	9.8 (7.6)	85	3.94 (1.97 to 5.91)^c^	<.001^d^
	Walking	218	9.0 (6.6)	85	3.46 (1.54 to 5.38)^c^	<.001^d^
	Heavy labor	218	0.8 (3.0)	85	0.48 (−0.13 to 1.10)	.12
**Work sitting accumulation**						
	Longest continuous sitting bout, min	218	138.1 (62.2)	85	−12.65 (−24.92 to −0.38)^c^	.04^d^
	Prolonged sitting, % of sitting	218	71.1 (20.2)	85	−8.74 (−14.14 to −3.34)^c^	.002^d^
**Before and after work activity, % of nonwork time (on work days)**						
	Sitting	218	47.2 (21.7)	85	−5.42 (−9.78 to −1.07)^c^	.02^d^
	Standing	218	18.1 (12.0)	85	0.46 (−2.33 to 3.25)	.75
	Moving^e^	218	34.7 (18.4)	85	4.96 (1.08 to 8.85)^c^	.01^d^
**Nonworkday activity, % of nonwork time (on nonwork days)**						
	Sitting	218	39.9 (20.5)	85	−4.68 (−8.80 to −0.57)^c^	.03^d^
	Standing	218	17.7 (11.6)	85	1.14 (−1.26 to 3.55)	.35
	Moving^e^	218	42.5 (18.9)	85	3.54 (−0.17 to 7.25)^c^	.06
**Desired activity, % of work time**						
	Sitting	216	41.2 (19.0)	82	−0.06 (−3.20 to 3.08)	.97
	Standing	216	28.2 (15.2)	82	2.17 (−1.60 to 5.94)	.26
	Moving^e^	216	30.6 (17.0)	82	−2.11 (−5.80 to 1.58)	.26
**Desired versus performed, absolute difference in % of time**						
	Sitting	216	37.7 (19.8)	82	−6.04 (−9.92 to −2.15)^c^	.002^d^
	Standing	216	18.2 (14.4)	82	0.70 (−3.15 to 4.54)	.72
	Moving^e^	216	21.7 (16.8)	82	−5.66 (−9.32 to −2.00)^c^	.002^d^
**Other outcomes**						
	Strategy usage (% strategies used ≥ “sometimes”)	215	41.3 (13.7)	82	7.32 (3.74 to 10.89)^c^	<.001^d^
	Knowledge score, 1 point=5 min incorrect	216	5.53 (8.19)	81	−0.33 (−2.63 to 1.96)	.78
	Control over sitting and standing (1-5)	216	2.94 (1.38)	82	0.06 (−0.21 to 0.33)	.66
	Support (1-5)	216	3.40 (1.20)	82	0.16 (−0.09 to 0.40)	.21
	Job performance (1-10)	212	7.74 (1.17)	80	−0.08 (−0.31 to 0.14)	.45
	Job satisfaction (1-10)	212	7.10 (2.17)	80	−0.05 (−0.45 to 0.35)	.81
	Self-rated health (1-5)	212	2.97 (1.02)	80	0.06 (−0.17 to 0.30)	.60
	Energy (1-4)	212	2.40 (0.68)	80	0.13 (−0.03 to 0.28)	.11
	Stress (1-4)	212	3.03 (0.78)	80	0.01 (−0.16 to 0.18)	.89

^a^Number of participants that completed the item on the baseline survey.

^b^Number of participants that completed the item on both surveys.

^c^Outcomes had a notable change (≥ the minimum difference of interest).

^d^Moving = walking + heavy labor.

^e^Outcomes are statistically significant at *P*<.05.

**Figure 2:**
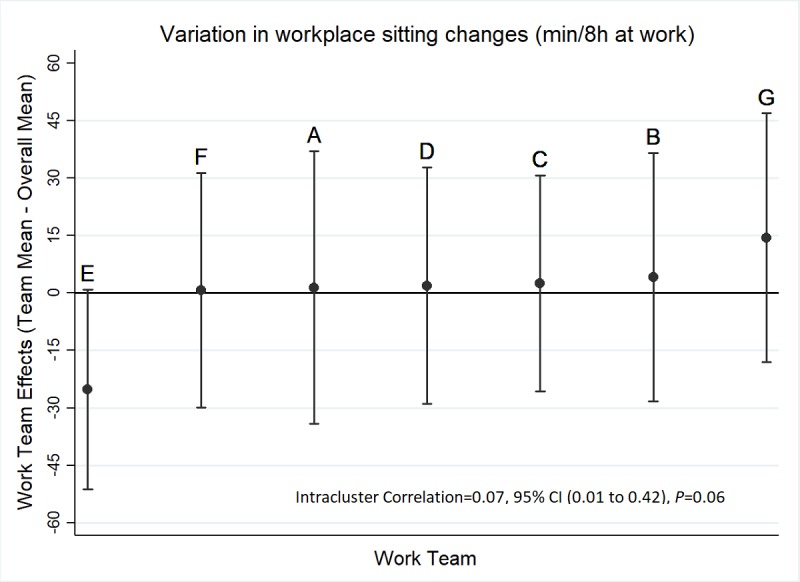
Best unbiased linear predictions of work team effects on workplace sitting changes (n=85 workers; n=7 teams) estimated from mixed models adjusting for baseline values with a random intercept for team.

Time outside of work was also most commonly spent sitting (mean 47.2% [SD 21.7%] of time before and after work and mean 39.9% [SD 20.5%] of time on nonwork days). However, by contrast with workplace time, moving reportedly occupied more time than standing outside of work hours. There was a notable difference between reported workplace behavior and desired levels of activity, with desired behavior (on average) involving less sitting and more standing and moving. This is illustrated, separately for each workplace, in Multimedia Appendix 2. When the difference between reported and desired sitting levels was considered at an individual level, 95% (205/216) of the staff wanted to sit less at work than they currently did.

The baseline survey results indicated that some of the potential impact outcome measures were more amenable to change compared with others, for which ceiling effects were a strong concern. On average, participants were using mean 41.1% (SD 13.7%) of the 13 activity-promoting strategies at least “sometimes” at baseline, leaving many strategies for participants to potentially adopt. In contrast, the mean levels of work performance and health indicators at baseline were typically well above midway on the scale on which they were measured, especially for stress and workplace support for reducing sitting. Respectively, 30% (63/212) and 17% (37/216) of participants had reported optimal levels of these outcomes at baseline.

#### After *BeUpstanding*

##### Workplace Sitting (Primary Outcome)

Over the course of the *BeUpstanding* intervention, mean workplace sitting time reduced significantly (*P*=.001) and substantially (−6.3%, 95% CI −10.1 to 2.5) among workers reporting activity at both surveys (n=85). This reduction amounts to −30.1 minutes (95% CI −48.4 to −11.8) over an 8-hour workday. Even when accounting for all 315 participants of both surveys by multiple imputation, workplace sitting still reduced significantly over the intervention (*P*=.02), but by a smaller extent (−3.9%, 95% CI −7.3 to −0.5 or −18.8 minutes per 8-hour workday, 95% CI −35.1 to −2.6). There was some evidence that the average change in workplace sitting varied from workplace to workplace with the random intercept for workplace being borderline significant (*P*=.06) and a moderate to strong degree of clustering observed (intracluster correlation=0.07, 95% CI 0.01 to 0.42) (Figure 2). The best-performing work team (E) had a workplace sitting reduction that was better on average by approximately 30 minutes per 8 hours at work. The remaining workplace-specific mean changes were very similar to the overall mean, with the largest deviation amounting to <15 minutes per 8 hours at work (Team G). Similar results were obtained even when accounting for the composition of each workplace in terms of worker age, sex, education, full-time equivalent, and job type (intracluster correlation=0.09, 95% CI 0.01 to 0.49, *P*=.05).

##### Secondary Outcomes

Changes in secondary outcomes are shown in Table 5. Statistically significant changes (all improvements) were observed in workplace moving, workplace walking, both of the sitting accumulation measures, moving outside of work, sitting on nonwork days, the deviation between desired and performed sitting and moving at work, and usage of the activity-promoting strategies. All of these changes were of a notable magnitude (≥MDI), or very nearly so in the case of sitting on nonwork days. Moving on nonwork days showed a notable improvement that did not reach statistical significance (*P*=.06). For the remaining nonsignificant activity-related outcomes, the 95% CIs indicated that substantial improvements were unlikely. The changes in activity outcomes indicated workplace sitting was primarily replaced with additional moving, specifically walking rather than heavy labor. This change resulted in there being a smaller gap between the behaviors participants reported performing and desiring, especially for sitting and moving. For those who completed both questionnaires, overall satisfaction with the program was high (80/86, 93% rated it ≥5/10).

Use (at baseline and follow-up) and changes in the use of individual behavioral strategies are shown in Multimedia Appendix 3. The strategies for which usage increased by >10% were as follows: “used an activity tracker/wearable device to track activity and/or sitting time” (+25.3%); “used prompts at my desk/around the office to remind me to stand up, sit less, move more” (+24.1%); “used the stairs instead of the lift” (+11.4%); and “stood up during a meeting” (+10.1%). The strategies most commonly used at least sometimes postintervention (partly due to high initial usage) were “walked to colleague rather than emailing/phoning” (97%); “ate my lunch away from my desk” (79%); and “went for a walk / did activity during the lunch break” (75%).

The average changes in the attitude, health, and work performance outcomes were both nonsignificant and very small. The observed change in self-reported energy levels (0.13, 95% CI −0.03 to 0.28) came the closest to a significant effect (*P*=.11), and at 0.18 SD fell just short of a “small” effect.

Intracluster correlations and responsiveness measures are reported in Multimedia Appendix 4. Workplace clustering effects were often negligible (<0.001) but were very strong (≥0.1) for some of the outcomes, including gap scores for sitting and standing, and workplace support for sitting and standing. Responsiveness varied widely between the different outcomes. Both SESs and SRMs indicated that the most responsive of the activity outcomes were workplace activity (especially sitting, moving, and walking), while the percentage of sitting accrued in prolonged bouts was the most responsive sitting accumulation outcome. Strategy usage had a similar responsiveness as per the activity changes. Of the attitudinal measures, gap scores for sitting and moving were the most responsive, while support for sitting and standing was the most responsive measure of job-related outcomes. Energy levels were the most responsive measure of health changes.

## Discussion

### Principal Findings

This study provides the first evidence of the feasibility of a sitting reduction intervention implemented by the workplace using a “train-the-champion” approach. The findings demonstrate that the beta (test) version of the *BeUpstanding Champion Toolkit* was feasible to implement by workplace champions. The impact findings from this pilot study also suggest that the adaptation of the *Stand Up Australia* intervention into its current, Web-based form (ie, *BeUpstanding*) was successful, as we saw significant, meaningful reductions in self-reported workplace sitting in staff who participated in the program evaluation.

A critical component for facilitating scale-up was the transfer of program delivery from the research team to a workplace champion, with support provided to the champion through a Web-based toolkit. The findings from this study suggest that this approach was successful, with the champions able to implement most or all of the intervention elements. Importantly, for scale-up, the time commitment required by the champions was relatively small, averaging 30-60 minutes per week across the study. The champions tended to be employed in job roles that facilitated conversations both up (to senior management) and down (to general staff). This ability to talk across levels has been previously identified as an important quality for workplace champions [39]. Additional research to identify and understand the key attributes of workplace champions will assist in providing guidance to organizations to inform their decisions on suitable candidates for the champion role.

All champions formed a wellbeing committee and involved members and work colleagues in the discussions of the strategies to promote increases in standing and moving more. Such support for the champion has been previously highlighted as key for helping maintain motivation for an initiative [39,40]. Notably, although the staff consultation workshop (and the associated collective decision on which strategies to implement as a team) is considered a core element of the program, one of the workplaces did not hold the workshop, 4 did not play the information video, and 2 did not choose any strategies as a team. These components (whose messages are then reinforced through the modifiable posters and email templates) are considered by the project team to be critical for raising awareness, building culture, and creating change (the 3 pillars of the *BeUpstanding* program). As such, an essential modification to the toolkit as part of the optimization process (Phase 3) will indicate the increased emphasis of the importance of the staff consultation session. Keeping track of implementation during the intervention and sending reminders to champions to complete critical steps will also be important.

Importantly, the toolkit enabled and empowered workers to choose and self-administer the changes that best suited their unique work team and environment, with a total of >30 different strategies selected by the work teams as part of their staff consultation workshop. The strategies chosen ranged across the hierarchy of control [10] and included environmental adaptation (eg, centralizing bins), substitution of work task activities (eg, standing instead of sitting during phone calls), and administrative changes (eg, no lunch at desk). Notably, many of the strategies chosen were low-cost or no-cost to the organization or individual. However, although we know the strategies chosen by the work team, we do not know their uptake and utilization as the strategy usage measured in the staff survey did not capture the wide range of strategies chosen by work teams. Larger and longer term studies with associated data collection of usage will facilitate the examination of which strategies are the most effective and sustainable, noting that some of the more novelty-based strategies (such as “dance-offs”) may still have a key role in raising awareness and creating momentum for culture change.

At baseline, participants reported sitting for nearly 80% of their workday on average, and nearly all participants (95%) had a desire to sit less. This finding is in line with studies that have used objective measures of activity in similar populations [7] and highlights the importance of targeting the desk-based workplace to address high levels of sitting time. Following the intervention, average self-reported workplace sitting decreased by 6.3% in those who completed both surveys. Assuming an 8-hour workday, this equates to an approximately 30-minute reduction per day in workplace sitting time. Although the response rate and the fact that the data were self-reported should be taken into consideration when interpreting the data, the findings are consistent with those achieved when interventions have been led by external research teams [34,41,42]. Results from the multiple imputation analyses were attenuated (3.9%) but were still statistically significant.

Despite the variety of workplaces included, there was minimal evidence that any workplace “underperformed.” The workplace with the greatest change (workplace E; ≈30 minutes per 8-hour workday greater change than the average across all teams) had recently installed sit-stand desks for their staff, a factor that is likely to have contributed to their relatively larger sitting time reduction [17,42]. Interestingly, despite it being a workplace-delivered program, significant beneficial and meaningful impacts were also seen for out of work sitting and moving time. Although these findings are preliminary, they reinforce the potential of the workplace as a key setting for addressing sedentary behavior more broadly.

Importantly, there were significant reductions in the indicators of prolonged sitting time. Prolonged, unbroken sitting time detrimentally impacts both cardiometabolic [43] and musculoskeletal health [44]; consequently, much of the messaging within the *BeUpstanding* program emphasizes the importance of regular postural shifts (alternating between sitting, standing, and moving). Findings from this feasibility trial suggest that the *BeUpstanding* program is effective in achieving relatively frequent changes in posture. However, it should be noted that most participants did not achieve their preferred levels of workplace sitting, with the gap between desired and reported sitting time at ≈31% on average at follow-up (compared with ≈37% at baseline). Additionally, approximately half of the participants did not agree that they had control over their sitting or standing. Such substantial changes, both in actual behavior and in perceived control, are unlikely to be achieved without associated system-level (eg, changes to work tasks and associated policies) and environmental-level (eg, incorporation of sit-stand workstations) supports, many of which are unlikely to be feasibly implemented within the short 3-month program timeframe. As part of the optimization process (Phase 3), the *BeUpstanding Champion Toolkit* will be revised to include more planning resources around longer term changes, and champions will be encouraged to repeat the program on an approximately annual basis, building on previous learning and successes. The revision will also include a sign-up page to recruit workplaces into the toolkit (access to the beta version was via a researcher-supplied login). Detailed evaluation of this process in the planned implementation trial (Phase 4) will be critical for informing the long-term success and large-scale dissemination of the program.

The health- and job-related outcomes did not change significantly following the *BeUpstanding* program. However, it is important to interpret these results cautiously in view of the study design and the survey response rates. The indices of responsiveness to change (SESs and SRMs) indicated limited responsiveness in all of the measures, due to both limited mean changes and high variability. The limited responsiveness could either be due to a genuine limited impact on these outcomes, measurement issues, or both. Notably, the phenomenon of ceiling effects was likely relevant to the limited change and may be an issue in any future implementation, with many outcomes having very favorable initial mean values with limited room to move. For example, 30% of participants had already reported the lowest possible stress level at baseline. While it is a common timeframe to assess behavior change, 3 months may not be a sufficient time to elicit measurable changes in health outcomes in this general worker population [45]; longer evaluations are needed. Concerning workplace performance, the lack of a sizable change beneficially or detrimentally was consistent with findings from systematic reviews [17,46]. Measurement may still be an issue, with the general measures used within the survey not tailored to workplace-specific tasks. Among the job- and health- related outcomes, the most promising indications of change concerned perceived support for sitting and standing, and self-reported energy levels; future evaluation with higher-quality measures is warranted. The use of performance and health indicator data routinely collected by the organization (eg, absenteeism rates, compensation claims, and employee engagement) may allow for a more robust evaluation of the impact of the *BeUpstanding* program, in both short and long term. Collaborating with workplaces to access such information and including business-relevant key performance indicators within the evaluation, will be important in helping to assess the business case for sitting reduction interventions such as *BeUpstanding*.

### Strengths and Limitations

A key strength of this study was the generation of practice-based evidence that will be used to inform the future optimization of the toolkit for wide-scale implementation. The work teams were purposively sampled, which provided input from a range of sectors, organizational sizes, and team locations. The sample was diverse in many regards but not necessarily representative, so generalizabilty is still a concern. For example, the sample was highly educated, had high baseline knowledge of the detrimental health impacts of excessive sitting, and predominantly had management responsibilities. Data were all self-reported, and response rates at follow-up were low, particularly for some work teams. This limits the quality of evidence gathered in the impact evaluation to corroborate the initial rigorous evaluation of the intervention prior to its adaptation into the Web-based *BeUpstanding* toolkit. Although this response rate is not untypical for this stage of research [47], future adaptations need to consider means of further engaging workers in the intervention. Technological advances mean that there are exciting opportunities for more regular and objective data capture options, such as through mobile phone platforms (eg, ResearchKit.org), or wearable activity tracker platforms (eg, Fitabase.com). These might help with both data collection and engagement. Further, data on any adverse impacts of the program were not collected as part of the staff survey, and detrimental impacts may have been missed.

### Conclusions

In summary, our findings indicate that this beta version of the *BeUpstanding Champion Toolkit* was feasible to implement using a “train-the-champion” approach and that the *BeUpstanding* program was effective in reducing prolonged workplace sitting and changing workplace practices without significantly or substantially detrimentally impacting the indicators of work performance. Besides significantly advancing the evidence base and providing proof of concept to inform larger implementation trials, this study has also begun to capture the practice-based evidence needed to inform ongoing, sustainable success. In addition to understanding how the program was implemented along with its impact, it is also important to know the acceptability of the program and champion and staff perceptions of the program, including facilitators and barriers to implementation. This, along with feedback regarding how the toolkit could be improved, will be used to inform the optimization of the toolkit to facilitate wide-scale uptake and implementation.

## Appendix

Multimedia Appendix 1 Baseline levels of the primary and secondary outcomes of the evaluable cases (n=85).

Multimedia Appendix 2 Mean of actual activity (a) and desired activity (b) at baseline within each workplace (n=216).

Multimedia Appendix 3 Strategies to sit less and move more at work that were used at least “sometimes.”

Multimedia Appendix 4 Intracluster correlation for change in primary and secondary outcomes and responsiveness measures.

## Abbreviations

MDI: minimum differences of interest
MVPA: moderate-to-vigorous physical activity
SES: standardized effect size
SRM: standardized response mean
