# Supplementary material for: Assessing the Feasibility and Pre-Post Impact Evaluation of the Beta (Test) Version of the BeUpstanding Champion Toolkit in Reducing Workplace Sitting: Pilot Study
Source: JMIR Form Res. 2018 Aug 28;2(2):e17. doi: 10.2196/formative.9343 (PMC6334681; doi:10.2196/formative.9343)
Supplement: Multimedia Appendix 1 [file formative_v2i2e17_app1.pdf]

Multimedia Appendix 2. Baseline levels of the primary and secondary outcomes of the evaluable cases (n = 85)[a].

	n	Mean	SD
**Work activity**			
Sitting	85	78.7	17.1
Standing	85	12.8	15.1
Moving	85	8.5	6.0
Walking	85	8.2	5.4
Heavy labor	85	0.3	1.3
**Work sitting accumulation**			
Longest continuous sitting bout, min	85	137.4	66.7
Prolonged sitting, % of sitting	85	70.3	20.2
**Before and after work activity,** % of nonwork time (on work days)			
Sitting	85	50.9	21.3
Standing	85	17.5	12.3
Moving	85	31.5	17.2
**Nonworkday activity,** % of nonwork time (on nonwork days)			
Sitting	85	45.7	21.6
Standing	85	17.0	11.0
Moving	85	37.3	18.4
**Desired activity,** % of worktime			
Sitting	84	41.6	20.6
Standing	84	30.0	16.7
Moving	84	28.4	16.7
**Desired versus performed**, absolute difference in % of time			
Sitting	84	37.2	21.0
Standing	84	18.8	15.8
Moving	84	20.4	15.8
**Other outcomes**			
Strategy usage (% of strategies used at least sometimes)	84	41.1	13.7
Knowledge score, 1 point=5 min incorrect	84	5.06	8.80
Control over sitting and standing (1-5)	84	3.05	1.35
Support (1-5)	84	3.68	1.08
Job performance (1-10)	82	7.75	1.16
Job satisfaction (1-10)	82	7.38	1.81
Self-rated health (1-5)	82	3.22	1.05
Energy (1-4)	82	2.45	0.77
Stress (1-4)	82	3.16	0.73

[a]Table reports mean and SD corrected for clustering (linearized variance estimation).
