# Supplementary material for: Assessing the Feasibility and Pre-Post Impact Evaluation of the Beta (Test) Version of the BeUpstanding Champion Toolkit in Reducing Workplace Sitting: Pilot Study
Source: JMIR Form Res. 2018 Aug 28;2(2):e17. doi: 10.2196/formative.9343 (PMC6334681; doi:10.2196/formative.9343)

Multimedia Appendix 4. Mean of actual activity (a) and desired activity (b) at baseline within each workplace (n=216).

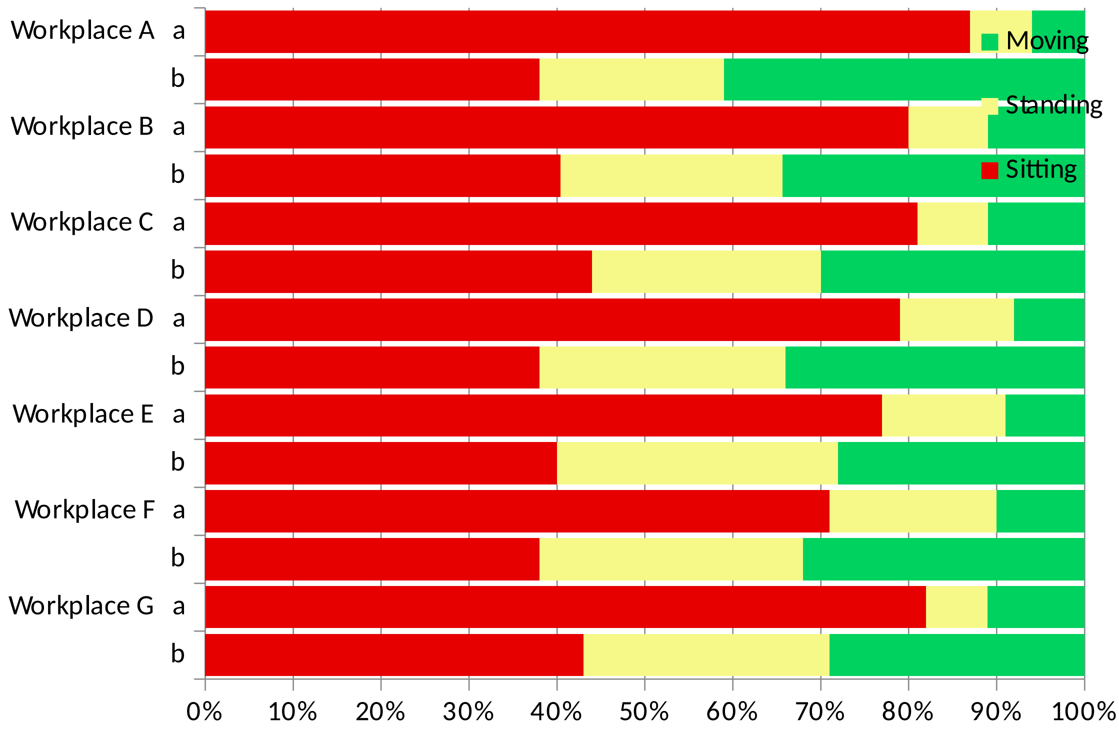

Supplement: Multimedia Appendix 2 [file formative_v2i2e17_app2.pdf]
