# Supplementary material for: Assessing the Feasibility and Pre-Post Impact Evaluation of the Beta (Test) Version of the BeUpstanding Champion Toolkit in Reducing Workplace Sitting: Pilot Study
Source: JMIR Form Res. 2018 Aug 28;2(2):e17. doi: 10.2196/formative.9343 (PMC6334681; doi:10.2196/formative.9343)
Supplement: Multimedia Appendix 3 [file formative_v2i2e17_app3.pdf]

Multimedia Appendix 1. Strategies to sit less and move more at work that were used at least "sometimes."

Strategy	**Baseline** **n=212** [a] n (%)	**Follow-up** **n=156**[a] n (%)	**Change** **n=79** [b] %
Stood up during a meeting	64 (30.2%)	65 (41.7%)	**+10.1%**
Stood up when the phone rang	86 (40.6%)	68 (43.6%)	+7.6%
Walked to colleague rather than emailing/phoning	206 (97.2%)	152 (97.4%)	0.0%
Ate my lunch away from my desk	152 (71.7%)	123 (78.8%)	+7.6%
Walked to printer further away	120 (56.6%)	94 (60.3%)	+3.8%
Walked to bathroom further away	110 (51.9%)	84 (53.8%)	+2.5%
Used the stairs instead of the lift	69 (32.5%)	65 (41.7%)	**+11.4%**
Went for a walk/did activity during lunch break	155 (73.1%)	117 (75.0%)	0.0%
Had a walking meeting	7 (3.3%)	17 (10.9%)	0.0%
Participated in activity class at my workplace	16 (7.5%)	15 (9.6%)	-3.8%
Used prompts at desk/around the office to remind me to stand up, sit less, move more	34 (16.0%)	53 (34.0%)	**+24.1%**
Used an activity tracker/wearable device to track activity and/or sitting time	60 (28.3%)	54 (34.6%)	**+25.3%**
Used a height-adjustable desk to alternate postures	59 (27.8%)	46 (29.5%)	+8.9%

[a]All participants who reported on strategies at baseline (n=212) or follow-up (n=156).
[b]Change within evaluable cases who reported on strategies at baseline and follow-up (n=79).
