# Supplementary material for: Assessing the Feasibility and Pre-Post Impact Evaluation of the Beta (Test) Version of the BeUpstanding Champion Toolkit in Reducing Workplace Sitting: Pilot Study
Source: JMIR Form Res. 2018 Aug 28;2(2):e17. doi: 10.2196/formative.9343 (PMC6334681; doi:10.2196/formative.9343)
Supplement: Multimedia Appendix 4 [file formative_v2i2e17_app4.pdf]

Multimedia Appendix 3. Intracluster correlation for change in primary and secondary outcomes and responsiveness measures.

Outcome	Intracluster correlation (ICC) ICC	Lower 95% Confidence Limit	Upper 95% Confidence Limit	SES[a]	SRM[b]
**Work activity**					
Sitting	0.068	0.007	0.416	-0.40	-0.36
Standing	0.073	0.010	0.390	0.19	0.18
Moving	<0.001	<0.001	<0.001	0.52	0.44
Walking	<0.001	<0.001	<0.001	0.53	0.40
Heavy labor	<0.001	<0.001	<0.001	0.16	0.17
**Work sitting accumulation**					
Longest continuous sitting bout, min	<0.001	<0.001	<0.001	-0.20	-0.19
Prolonged sitting, % of sitting	<0.001	<0.001	<0.001	-0.43	-0.37
**Before and after work activity**					
Sitting	<0.001	<0.001	<0.001	-0.25	-0.26
Standing	<0.001	<0.001	<0.001	0.04	0.01
Moving	<0.001	<0.001	<0.001	0.27	0.28
**Nonworkday activity**					
Sitting	<0.001	<0.001	<0.001	-0.23	-0.21
Standing	<0.001	<0.001	<0.001	0.10	0.07
Moving	<0.001	<0.001	<0.001	0.19	0.18
**Desired activity**					
Sitting	<0.001	<0.001	0.000	0.00	0.00
Standing	0.089	0.004	0.689	0.14	0.12
Moving	<0.001	<0.001	0.995	-0.12	-0.12
**Gap scores[c]**					
Sitting	0.132	0.022	0.507	-0.30	-0.34
Standing	0.203	0.036	0.632	0.05	0.04
Moving	0.036	0.002	0.481	-0.34	-0.33
**Other outcomes**					
Strategy usage (% of strategies)	0.002	<0.001	1.000	0.53	0.44
Knowledge score, 1 point = 5 min error	0.028	<0.001	0.757	-0.04	-0.03
Control over sitting and standing 1-5	0.079	0.006	0.533	0.04	0.05
Support 1-5	0.183	0.035	0.584	0.13	0.14
Job performance 1-10	<0.001	<0.001	<0.001	-0.07	-0.08
Job satisfaction 1-10	<0.001	<0.001	<0.001	-0.02	-0.03
Self-rated health 1-5	0.039	<0.001	0.972	0.06	0.06
Energy 1-4	0.004	<0.001	1.000	0.18	0.18
Stress 1-4	0.080	0.004	0.635	0.02	0.02

[a]Standardized effect size (SES) = mean change/SD at baseline. Mean change and baseline SD were calculated as reported in Table 5. Larger absolute values of SES indicate better responsiveness to change.

[b]SRM = standardized response mean: change/SD of change. Mean and SD of change scores were calculated using linearized variance estimation. Larger absolute values of SRM indicate better responsiveness to change.

[c]Gap scores = absolute value of difference between desired and performed activity at work.
